# Proteasome-dependent senescent tumor cells mediate immunosuppression through CCL20 secretion and M2 polarization in pancreatic ductal adenocarcinoma

**DOI:** 10.3389/fimmu.2023.1216376

**Published:** 2023-06-15

**Authors:** Mengwei Wu, Jiashu Han, Hao Wu, Ziwen Liu

**Affiliations:** ^1^ Department of General Surgery, Peking Union Medical College Hospital, Chinese Academy of Medical Sciences & Peking Union Medical College, Beijing, China; ^2^ Chinese Academy of Medical Sciences and Peking Union Medical College, Beijing, China

**Keywords:** pancreatic cancer, senescence, tumor microenvironment, tumor-associated macrophages, prognosis

## Abstract

The outcome of pancreatic ductal adenocarcinoma (PDAC) remains poor due to few therapeutic options available and challenges with precision therapy to target each tumour’s specific characteristics. In this study, a biologically meaningful patient stratification-prognostic model with therapeutic suggestion value based on tumor senescence was developed and validated in multiple independent cohorts. Further mechanistic investigation based on single-cell transcriptomic data and *in vitro* experiments revealed that complement derived from non-senescent tumor cells stimulates M1 differentiation and antigen presentation, while senescent tumor cells secrete CCL20 to favor immunosuppressive M2 polarization. Also, senescent phenotype depends on proteasome function, suggesting that high-risk, high-senescence patients may benefit from proteasome inhibitors, which reverse senescence-mediated resistance to conventional chemotherapy and improve outcome. In conclusion, the current study identified senescence as a tumor-specific, hazardous factor associated with immunosuppression in PDAC. Mechanistically, senescence abrogates complement-induced M1 activation and antigen presentation, and upregulates CCL20 to favor M2 polarization. The senescence-related risk model is prognostic and therapeutic-suggestive. In light of the reliance of senescent cells on proteasomal functions, proteasome inhibitors are promising agents for high-risk patients with senescent PDAC.

## 1 Introduction

Pancreatic ductal adenocarcinoma (PDAC) is a highly lethal disease due to generally poor response to radiotherapy, gemcitabine-based or mFOLFIRINOX chemotherapy, or immunotherapy (1). Some patients with PDAC are rapidly progressive within months, while some treatment-responsive patients persist for years. Such disparity in prognosis is unaccounted for by the conventional TNM and pathohistological classifications (2). A molecular-centered approach dwelling into the biological characteristics behind these clinical behaviors would not only allow more accurate patient stratification, but also suggest precise targeted therapy.

Marked by highly dysplastic tumor microenvironment and complex heterogeneity, much effort has been put into the molecular subtyping of patients with PDAC (3, 4). Single gene alteration in BRCA1/2, BRAF, ERBB2s, PIK3CA, ATM, and MMR has advantage in therapeutic suggestion, but limited application value as less than 5% of patients with PDAC have such targetable mutations (5). The Waddle signature, based on whole genomic features such as copy number and chromosomal structural variation, divided patients with PDAC into four subtypes: stable, locally rearranged, scattered, and unstable (sensitive to platinum and PARP inhibitors) (5). Transcriptomic-based subtyping provided more comprehensive information. Earliest attempt proposed the four subtypes, exocrine, classical, and quasi-mesenchymal, based on expression of digestive enzymes, epithelial adhesion genes, and mesenchymal genes (6). The most widely accepted classical-basal defined tumor-specific classical (GATA6, PDX1, and HNF1A) and more malignant basal-like subtypes (7). Further analysis took the tumor microenvironment into consideration and identified four subtypes with distinct stromal and immune characteristics: basal, stromal activated, desmoplastic, and the immune-classical subtypes (8). Application of laser capture micro dissection allowed the characterization of intratumoral spatial heterogeneity (9). However, all of these schemes gave no explanation on the reason and biological-molecular mechanisms underlying these different subtypes.

Cellular senescence is an induced state of growth arrest in response to stress and aging, presented under both physiological and pathological conditions (10). In contrast to uncontrolled cell death from noxious stimuli, cellular senescence is a regulated process associated with extensive epigenetic-transcriptomic-metabolic reprogramming and activity of multiple signal transduction pathways (11). Interestingly, senescence of cancer cells has been reported to have context-dependent functions, with both tumor-promoting and suppressive potentials (12). Senescent cells are marked by senescence-associated secretory phenotype (SASP) of cytokines (IL-1α, IL-1β, IL-6, IL-8), chemokines (CCL2, CCL5, CXCL1), growth factors (HGF, EGF, TGFα), and matrix-remodeling enzymes (MMP1, MMP3) (13). SASP has been reported to aid immunosurveillance through CCL2+ macrophages and CD4+ T cells, CXCL14/IGFBP3 mediated immune cell infiltration, and innate effector function through p53 induced apoptosis (14). However, SASP has also been associated with immunosuppression in terms of JAK2/STAT3 pathway signaling, chronic inflammation, and M2 macrophages that negatively engage with cytotoxic T and NK cells (15). The effect of cancer senescence on the clinical course of PDAC and its role in microenvironment remodeling remain obscure.

We hereby developed a senescence-based prognostic model that not only stratify patients by risk and survival probability, but also suggest subsequent therapeutic options. We further sought to elucidate the context-dependent effect of SASP specifically in PDAC, focusing on the exact axis of immunosuppression, leading to potential targetable points to break the negative feedback loop and precisely improve immunotherapy response in conjugation with ICB and/or cellular therapies.

## 2 Materials and methods

### 2.1 Data collection and processing

Transcriptomic data and clinical information of patients with PDAC were acquired from The Cancer Genome Atlas (TCGA, https://portal.gdc.cancer.gov/), the PACA-AU and the PACA-CA databases (ICGC Data Release 28, https://dcc.icgc.org/). A combined cohort of TCGA and GTEx normal samples recomputed by UCSC Xena as log normalized count matrix was obtained for the identification of DEGs in PDAC (UCSC Xena, https://xenabrowser.net/). For the TCGA dataset, raw count expression data was downloaded from the TCGA Data Portal with the TCGAbiolinks R package (16). For the PACA-CA dataset, raw count expression data was downloaded from ICGC (ICGC Data Release 28, https://dcc.icgc.org/projects/PACA-CA). Raw count expression data was then transformed into normalized count with the Deseq2 R package and VST function (17). Microarray gene-expression data of PACA-AU was downloaded from the ICGC database (ICGC Data Release 28, https://dcc.icgc.org/projects/PACA-AU). The data resources used in this study were summarized in **Table S1**. All the R packages used in this study run in the R software (version 4.1.2, https://www.r-project.org). Single-cell transcriptomic data of cancerous and normal pancreatic tissue were obtained from the Genome Sequence Archive under project PRJCA001063, under the accession number of GSA: CRA001160 (18). 554 patients with PDAC were retrospectively enrolled and analyzed in this study: the TCGA, PACA-AU and PACA-CA cohorts each included 141, 231 and 182 patients with detailed clinical information, survival follow-up, and complete genomic and transcriptomic data, respectively (**Table S2**).

### 2.2 Patient stratification

The “PlotPCA” function of the Deseq2 R package generated principal component analysis (PCA) plots. Gene set enrichment analysis (GSEA) revealed differences in pathway activities and biological characteristics between cancerous and normal pancreatic tissues. Genes that are differential expressed (DEGs), defined as false discovery rate < 0.05 and the |fold change| > 1, between cancer and normal pancreatic tissues were identified with the “Deseq2” R package based on raw count data obtained from the combined cohort of TCGA and GTEx normal samples recomputed by UCSC Xena. DEGs for arrays samples were identified with the “Limma” R package under the same criteria for significance (19). A senescence gene set was manually curated from published literatures and databases, including Kyoto Encyclopedia of Genes and Genomes database, the Gene Ontology database, the Reactome database, the Molecular Signatures Database, the Csgene database (20) and Cellage (21) (**Table S3**) and overlapped with DEGs in PDAC. The resulting genes were used to cluster patients with PDAC using “NMF” R package (22). The R package “ComplexHeatmap” displayed the expression of key senescent genes along with clinicopathological characteristics to compare between the two groups (23). Kaplan-Meier (K-M) method and log-rank test with the R packages “survival” and “survminer” were employed to generate survival curves and determine the prognostic value. Enrichment analysis was performed with DAVID, with KEGG, GO, and REACTOME. GSEA analysis was performed with clusterProfiler 4.0 R package (24).

### 2.3 Characterize immunosuppression of senescence

The abundances of infiltrating immune cells of each subtype, stromal cells, and cancer cells were inferred from bulk transcriptomic data with the “IOBR” R package (25) and displayed as an average of each group in heat map generated with “ComplexHeatmap”. Pathway activities were calculated with the ssGSEA algorithm in “GSVA” R package (26), using previously annotated pathways obtained from the Molecular Signatures Database, including “trafficking of immune cells to tumors”, “priming and activation”, “CD8 effector functions and exhaustion”, “immunogenic cell death”, “MHC class II”, “antigen processing and presentation”, “phagocytosis”, “bad angiogenesis”, “hypoxia”, “exosome release”, and “SASP”. The tumor purity score, immune infiltration level, and stromal content were evaluated *via* the “ESTIMATE” algorithm (27). The R package “Maftools” was used to calculate tumor mutation burden (TMB) from whole-exome sequencing (WES) data (28). The correlations between SASP activity (determined with ssGSEA) and the abundances of each cellular subtypes (inferred using the “IOBR” R package) were calculated by correlation analysis and displayed on a lollipop diagram generated in PRISM.

Single cell transcriptomic data was analyzed. Briefly, the gene-barcode matrix provided was read into the Seurat R toolkit (29) for subsequent processing and analysis; provided annotations on sample information were selectively adopted. Quality control was performed by removing cells with low quality (less than 200 genes and mitochondrial genes above 10%). Genes expressed in less than 3 cells are also removed. Next, principle component analysis with the 3000 highly variable genes clustered all cells into 9 major cell types, each with characteristically expressed markers displayed in feature plots. Ductal 2 cells, macrophages, and T cells were individually re-clustered for subsequent analysis. Ductal 2 cells were annotated as senescent and non-senescent tumor cells based on CDKN2A expression, or CCL20+ versus CCL20- tumor cells based on CCL20 expression. Subtype proportion were defined as the number of cells in each subset divided by the total number of cells in the major cell type. T-test was used to compare differences in proportion, and Pearson’s analysis was used to determine correlation between cell subtypes accounting for the sample from which each cell was collected. The proportion of senescent tumor cells was used to stratify patients into sene-high and sene-low groups by median value. AUCell was employed to analyze ‘gene set’ activity with the “Area Under the Curve” (AUC) method to calculate whether a critical subset of the input gene set is enriched within the expressed genes for each cell, employing consensus antigen presentation gene modules from GSEA (30). Cellular interaction was analyzed with the CellChat R package (https://github.com/sqjin/CellChat) base on ligand-receptor interaction database CellChatDB 2.0. Differential expression of ligands and receptors for each cell type or subtype infer cellular communications. The probability of interactions are visualized through heat maps, weighted directed circular graphs, and hierarchical plots (31).

### 2.4 Construction and validation of model

The PACA-AU dataset was employed as the primary construction dataset in which the model was first established, and the TCGA and PACA-CA datasets served as external validation datasets for further extrapolation and fitting of the model. The “WGCNA” R package (32), first under default parameters, identified the key gene modules (pink) with the highest absolute module significance and strongest association with C2. Least absolute shrinkage and selection operator (LASSO) regression, with the optimal penalty parameter and a minimum 10-fold cross-validation, was applied to remove the multicollinearity among these genes and to identify the most valuable prognostic genes. Finally, 12 genes were included in the final model, with Cox regression giving out a linear predictor equation and risk score for survival probabilities for patient stratification.

The robustness of the model was evaluated by calibration at different timepoints through time-dependent receiver operating characteristic (ROC) curves made with the “survivalROC” R package. Moreover, patients are stratified into high and low risk groups by an optimal cutoff value according R package “maxstat”, and generated survival curves *via* the Kaplan-Meier (K-M) method and log-rank test with the R packages “survival” and “survminer”. Collectively, area under the curve (AUC) of the ROC curve, Brier Score, and K-M survival analysis were considered when evaluating the model.

We further constructed a nomogram to improve the predictive power of this model. Firstly, The “Ezcox” R package was used to perform univariate proportional hazards (Cox) regression analysis to identify survival-related clinical characteristics (including the risk score). Secondly, multivariable Cox regression model screen out independent predictive characteristics, which were visualized with a forest plot from the “survminer” R package. Thirdly, a stepwise multivariate Cox regression combined these factors to construct the final nomogram, including Sene-index, grade, N stage, and AJCC stage. The nomogram was further evaluated for predictive probability of survival at different timepoints in patients with PDAC using the “survival” R package, along with calibration curves and Brier Score for evaluation of accuracy. Decision curve analysis (DCA), performed with “ggDCA” R package. is a suitable method for evaluating alternative diagnostic and prognostic strategies that has advantages over other commonly used measures and techniques. The nomogram was internally validated using the Bootstrap method with b=100 by “plotCalibration” function of “riskRegression” R package.

### 2.5 Exploration of therapeutic sensitivities

“Oncopredict” R package, a tool based on the Genomics of Drug Sensitivity in Cancer (GDSC) databases, was employed to predict the half-maximal inhibitory concentration (IC50) of common therapeutic drugs (33), which were then compared between the high- and low-risk groups by unpaired t-test; the results were displayed using the “ggpubr” R package. The correlations between IC50 values and the risk score were analyzed with correlation analysis and displayed on bubble plot generated in PRISM.

### 2.6 Experimental validations

#### 2.6.1 Cell cultures

The pancreatic cancer cell lines PANC-1, BXPC-3, and ASPC-1 were obtained from ATCC and kept under recommended conditions of RPMI 1640 medium (Thermo Fisher, 11875093) with1% penicillin and streptomycin (Thermo Fisher, 15070063), and 10%, 10% and 20% fetal bovine serum (Thermo Fisher, 10099), respectively. The human monocyte cell line THP-1 was similarly maintained. Treatment with 12-O-tetradecanoylphorbol-l3-acetate (PMA) for 24 h at concentration of 10 ng/ml mediated monocyte to macrophage activation, serving as non-differentiated control. M1 and M2 differentiation were maintained with 50 ng/ml recombinant human IFN-γ (Peprotech, 300-02) or 25 ng/ml of recombinant human IL-4 (Peprotech, 200-04), respectively. Cell cultures were regularly tested to ensure mycoplasma-free with MycoAlert mycoplasma detection kits (Lonza, LT07) and sent for multiallelic variable number of tandem repeats sequencing to rule out cross-contaminations.

#### 2.6.2 Cellular senescence

Pancreatic cancer cell lines were seeded into 6-well plates at 50% confluence, rested overnight before exposed to 100 nm doxorubicin (MCE, 23214-92-8) for 48 h, and maintained for 15 days for the induction of cellular senescence. Conditioned media were collected at day 7 and 9 after Dox treatment for quantification of CCL20 secretion by ELISA (Abcam, ab269562).

#### 2.6.3 mRNA quantification

RT-qPCR was employed to quantify mRNA expressions. Briefly, total RNA was obtained with RNeasy kit (Qiagene 74004) following manufacturer’s instruction. cDNA was synthesized with the M-MLV reverse transcriptase kit (Thermo Fisher, 28025013). RT-qPCR reaction was performed in a final volume of 20 μ containing 12 μl TaqMan^®^ Universal PCR Master Mix, 5 μl H2O, 1 μl of forward and reverse primers, and 1 μl cDNA (approximately 10ng/μl). The reaction was put in an ABI PRISM^®^ 96-Well Optical Reaction Plate under the standard thermal cycling conditions by ABI PRISM^®^ 7000 Sequence Detection System (TaqMan^®^): initial 50°C for 2 min and 95°C for 10 min followed by 40 cycles at 95°C for 15 sec and 60°C for 1 min were used. All reactions were performed in three duplicates. The following primers are used for qPCR reactions:

**Table:** 

Gene	Forward sequence	Reverse sequence
p16INK4A	CTCGTGCTGA TGCTACTGAGGA	GGTCGGCGCAGT TGGGCTCC
P21	AGGTGGACCT GGAGACTCTCAG	TCCTCTTGGAGA AGATCAGCCG
IL-1a	TGTATGTGAC TGCCCAAGATGAAG	AGAGGAGGTTG GTCTCACTACC
CCL20	AAGTTGTCT GTGTGCGCAAATCC	CCATTCCAGAAA AGCCACAGTTTT
20S	TTCTGGCTCC TTGGCAGCAATG	CAGGTCGTTGA AGATGCCAGCT

#### 2.6.4 Drug resistance

Cells seeded in 96-well-plates were rested overnight and treated with gemcitabine (MCE, 95058-81-4), paclitaxel (MCE, 33069-62-4) and/or bortezomib (MCE, 179324-69-7) over a wide spectrum of concentrations. MTT assays (Thermofisher Scientific) were performed according to the manufacturer’s instruction to evaluate cell viability.

## 3 Results

### 3.1 Senescence in PDAC

**Figure 1:**
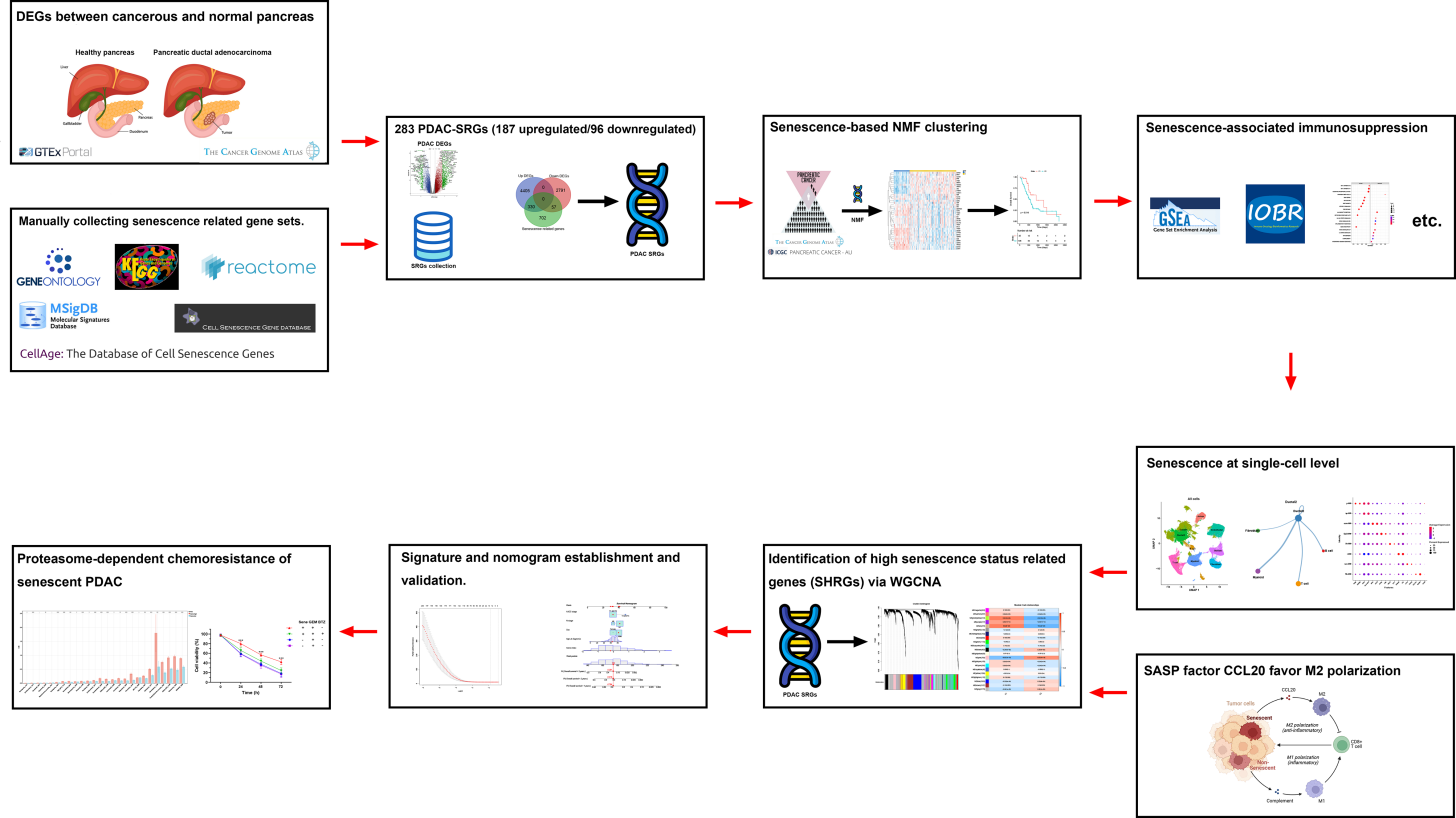
Overview of study design and flowchart.

**Figure 2:**
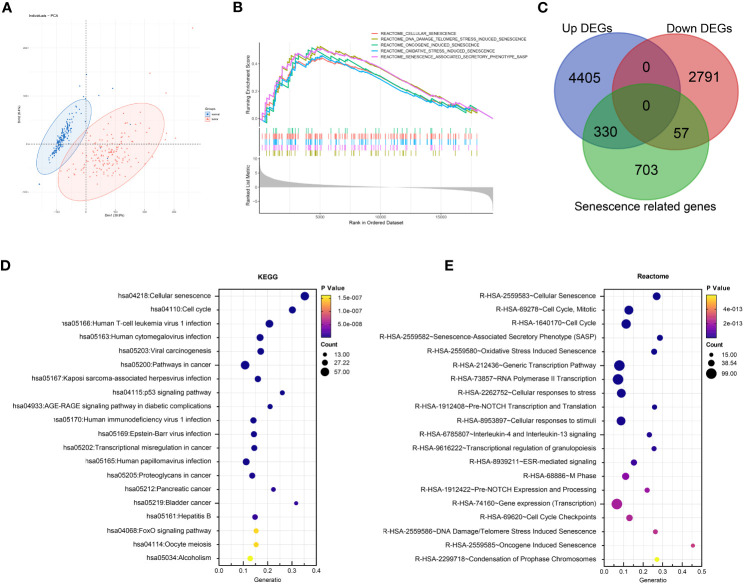
Senescence in tumorigenesis of PDAC. **(A)** Principal component analysis (PCA) of normal pancreatic tissue (blue) and cancerous PDAC (red). **(B)** Gene set enrichment analysis (GSEA) comparing the activity of REACTOME senescence gene sets in cancerous versus normal tissue. **(C)** Venn diagram displaying the number of senescence related genes up- and down-regulated in cancerous versus normal tissue. Enrichment analysis of DEGs between cancerous and normal tissues with **(D)** Kyoto encyclopedia of genes and genomes (KEGG) and **(E)** REACTOME databases respectively.

A flowchart of the study is described in **Figure 1**. Using the transcriptomic data of PDAC and paired control from the TCGA and GTEx dataset, we first confirmed distinct transcriptomic profiles between cancerous and normal tissue, representing biological reprograming associated with tumorigenesis (**Figure 2A**). Further GSEA analysis with REACTOME gene sets suggested high senescence in cancerous compared to normal tissue, with mutually agreeing result from multiple senescence pathways including cellular senescence (NES = 1.5259, P < 0.001), DNA damage/telomere stress induced senescence (NES = 1.5986, P = 0.007), oncogene induced senescence (NES = 1.4341, P = 0.059), oxidative stress induced senescence (NES = 1.5104, P < 0.006), and senescence associated secretory phenotype (NES = 2.274, P < 0.001) (**Figure 2B**). Considering senescence as a key feature for malignancy, we subsequently created a curated senescence gene set (**Table S3**) based on published literatures and databases, including Kyoto Encyclopedia of Genes and Genomes database, the Gene Ontology database, the Reactome database, the Molecular Signatures Database, the Csgene database and the CellAge database. After the removal of repetitive genes, the resulting senescence gene set included 1090 unique genes, among which 330 are up-regulated and 57 down-regulated in cancerous versus normal pancreas (**Figure 2C**, **Table S3**). These 387 PDAC-senescence genes are further analyzed by pathway enrichment analysis in the KEGG and REACTOME databases, confirming their key correlation with the biological process of senescence (**Figures 2D, E**).

**Figure 3:**
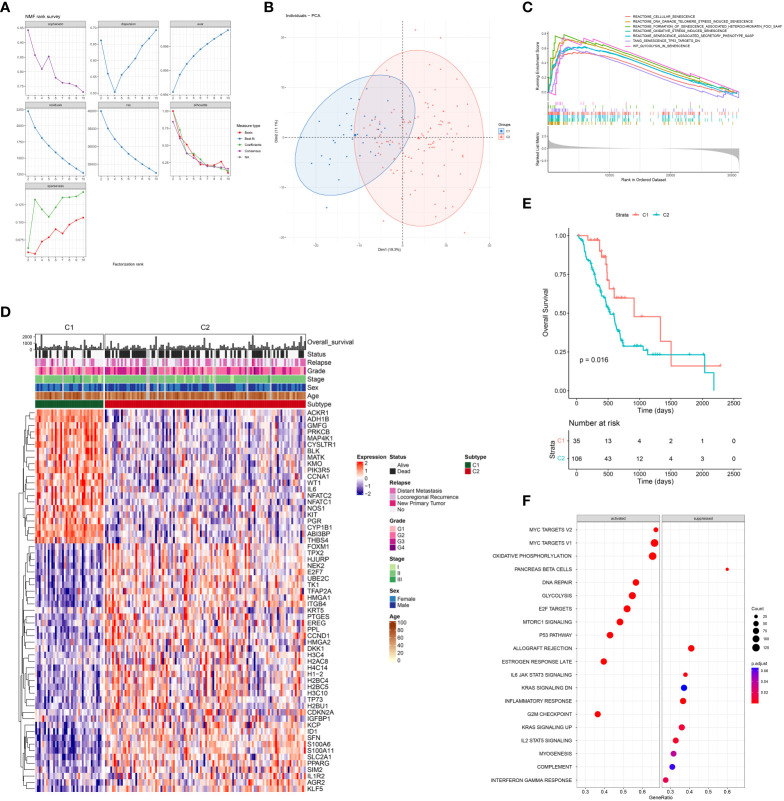
Senescence-based PDAC patients’ stratification by NMF clustering in TCGA. **(A)** Optimization of parameters employed by non-negative matrix factorization (NMF) clustering based on differentially expressed senescence related genes. **(B)** Principal component analysis (PCA) of C1 (blue) and C2 (red). **(C)** Gene set enrichment analysis (GSEA) comparing the activity of REACTOME senescence gene sets in C2 versus C1. **(D)** Heatmap showing differential expression of senescent genes between C1 and C2, along with distribution of clinicopathological characteristics including overall survival (OS) and status of death event, relapse, grade, stage, sex, and age. **(E)** Survival analysis of C1 and C2 with Kaplan-Meier curve. **(F)** Enrichment analysis of DEG between C2 and C1 with Gene-ontology: Biological Process (GO-BP).

To further elucidate the prognostic role of senescence in PDAC, we performed NMF clustering of TCGA dataset based on these 387 genes (**Figure 3A**), giving rise to two populations of patients, noted C1 and C2 respectively, with distinct transcriptomic profiles as clear separation by PCA analysis (**Figure 3B**). Senescence was characterized with GSEA analysis to be significantly higher in C2 (**Figure 3C**). Senescence related genes that are differential expressed between C1 and C2 (**Figure 3D**), denoting C2 as the high-senescence and C1 as the low-senescence group. Notably, the two groups had comparable clinicopathological features in terms of age, sex, stage, grade and status of relapse (**Figure 3D**), highlighting senescence as an independent factor intrinsic to tumor biology. Kaplan-Meier analysis revealed worse outcome of C2 as rapid drop in survival curve (log-rank test, P = 0.016; mOS 11 months versus 15 months, **Figure 3E**), suggesting a pro-tumor, hazardous role of senescence in PDAC. As PCA suggested distinct transcriptomic profiles, we further compared biological characteristics between the two subtypes, revealing significantly more growth and cell division, but suppressed immune activities in C2 (**Figure 3F**). The aforementioned results are entirely reproducible in PACA-AU (**Figure S1**).

### 3.2 Senescence-mediated immunosuppression

**Figure 4:**
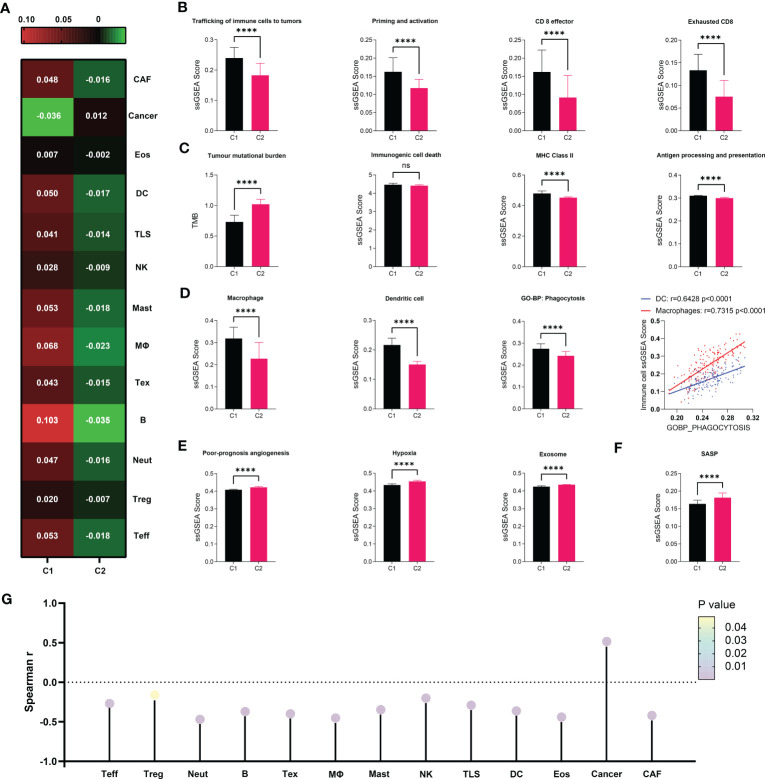
Immunosuppressive microenvironment associated with senescence in TCGA. **(A)** cell abundance inferred by IOBR are displayed in heat map to allow comparison between C1 and C2. **(B–F)** Comparison of immune cell abundance (macrophage and dendritic cell) and immunological activities in terms of pathways including “trafficking of immune cells to tumors”, “priming and activation”, “antigen processing and presentation”, “CD8 effector”, “CD* exhaustion”, “poor-prognosis angiogenesis”, “hypoxia”, and “exosome” defined by IOBR, or “immunogenic cell death”, “MHC class II”, “phagocytosis” and “SASP” defined by ssGSEA. Statistical analysis was performed with unpaired student t-test (****p<0.0001; ns, not significant). Correlation between DC or Macrophage and Phagocytosis was analyzed by Pearson correlation analysis. **(G)** Correlation between SASP as defined by ssGSEA and immune cell abundance was evaluated through Spearman correlation analysis, visualized as lollipop diagram.

Given the importance of anti-tumor immunity in tumorigenesis, prognosis and therapeutic response of PDAC, and the strong correlation between immune and senescence as we previously discovered (**Figure 3F**), we further compared the immune microenvironment between C1 and C2, aiming to characterize the immunological effects of senescence. TCGA (**Figure 4**) and PACA-AU (**Figure S2**) have agreeing results. Comparison of cellular signatures suggested an immune-desert microenvironment in C2, with less immune and stromal cells but more cancer cells (**Figure 4A**), consistent with the low immune activity as previously portrayed. The course of an adaptive antitumor immunity requires the following components: 1) antigen availability and release from tumor cells, 2) activation and antigen presentation by antigen presenting cells (APCs), and 3) effector cell chemotaxis, activation and effector function, and finally exhaustion. To find out which of these steps went wrong in senescent tumors, we went through each of these processes step by step. T cell functions are significantly impaired by high senescence, supported by impaired trafficking, priming/activation, effector function (**Figure 4B**) along with decreased number of Teff and even Treg and Tex (**Figure 4A**). Tracing backward, there was no evidence for decreased antigen availability as the reason behind immunosuppression in C2, as C2 seemed to have higher antigen load due to higher tumor mutation burden (TMB) and comparable immunogenic cell death (**Figure 4C** 1-2). However, C2 had lower MHC II, macrophages, dendritic cells, and phagocytosis (**Figure 4C, D**), suggesting impaired antigen presentation. The importance of phagocytosis in APC has been further confirmed to be in strong correlation with both APCs (**Figure 4D**). We further explored the reasons behind APC suppression, and found a variety of potential causes including bad-angiogenesis, hypoxia, and tumor-derived exosome (**Figure 4E**). Markedly, senescence-associated secretory phenotype (SASP), which has gained much attention as the key to senescence-mediated immunosuppression, is also higher in C2 compared to C1 (**Figure 4F**). Considering the importance of SASP, we further evaluated its correlation with infiltrating immune cells, showing a significant negative correlation with all immune and stromal cells, but positively with cancer cells (**Figure 4G**). Among key SASP factors, CCL20 was highly expressed in C2 versus C1 (**Figure S3A–C**), and correlated with poor prognosis of PDAC (HR, 1.10; 95% CI, 1.03–1.17; P = 0.004) and multiple cancer types. PDAC patients with high versus low expression of CCL20 were clearly separated on KM (log-rank test, P = 0.0021; mOS 532 days versus 2084 days, **Figure S3D, E**).

### 3.3 Mechanism of antigen presentation impairment

**Figure 5:**
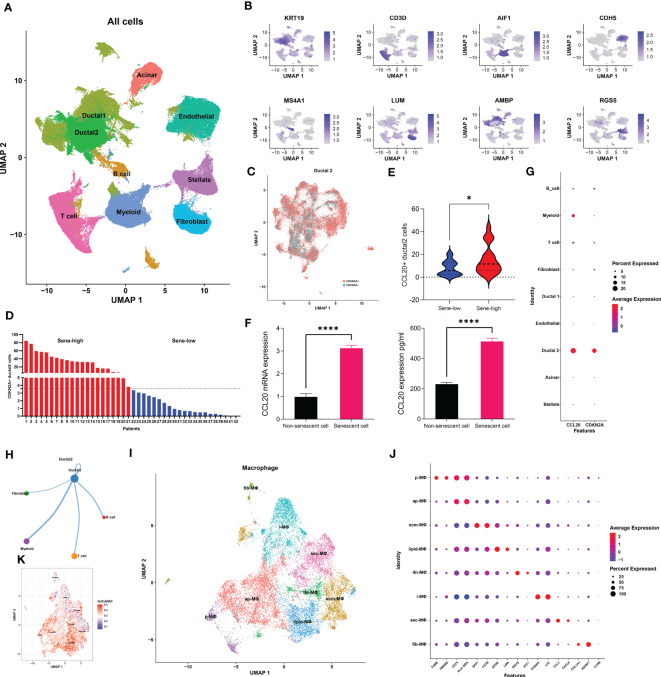
Senescent tumor cell derived CCL20 favors M2 polarization. **(A)** Dimension reduction and major cell types annotation visualized by UMAP. **(B)** Visualization of cell-type specific markers on feature plot. **(C)** Sub-clustering of Ductal 2 cells and definitional of CDKN2A expression +/-. **(D)** Stratification of patients into two groups with high or low senescence based on medium cutoff of percentage CDKN2A positive Ductal 2 cells. **(E)** Comparison of percentage Ductal 2 cells positive for CCL20 expression. **(F)** Quantification of CCL20 in PANC-1 cell under doxorubicin-induced senescence versus control with qPCR and ELISA. **(G)** Expression of CDKN2A and CCL20 in each major cell types. **(H)** The number of cellular interactions between each major cell types as determined by CellChat. **(I)** Further clustering of macrophages projected on UMAP into 8 distinct clusters. **(J)** Expression of specific markers of each macrophage subset. **(K)** Quantification of antigen presentation capacity in terms of activity of the “antigen processing and presentation pathway” determined by AUCell. * p<0.05, **** p<0.0001.

We next utilized single-cell transcriptomic data to gain insight on the mechanism by which senescent tumor cells and CCL20 suppress antigen presentation and APC functions. We first annotated 9 major cell types based on markers previously published by Wenming Wu and other literatures: KRT9 for malignant ductal 2, CD3D for T cells, AIF1 for myeloid cells, CDH5 for endothelial cells, MS4A1 for B cells, LUM for fibroblasts, AMBP for benign ductal 1, RGS5 for stellate cells, and insulin B for acinar cells (**Figures 5A, B**, **Figure S4A**). To characterize senescence status of tumor cells, we re-clustered ductal 2 cells and annotated all ductal 2 cells as CDKN2A+ and CDKN2A-, or senescent versus non-senescent tumor cells (**Figure 5C**). Then, based on the proportion senescent tumor cells, we stratified patients into sene-high and sene-low (**Figure 5D**). As CCL20 was identified to be the key SASP factor in PDAC, we compared CCL20 secretion between sene-high and sene-low patients, and found higher CCL20 associated with senescence (**Figure 5E**). Upregulation of CCL20 secretion in senescent tumor cells was further validated by RT-qPCR quantification of mRNA expression and ELISA quantification of protein (**Figure 5F**). Observing the expression pattern among all major cell types, the expression of CDKN2A and CCL20 are highly correlated and ubiquitous to malignant ductal 2, suggesting the key role of senescent tumor cells as secretor of CCL20 in the tumor TME (**Figure 5G**).

**Figure 6:**
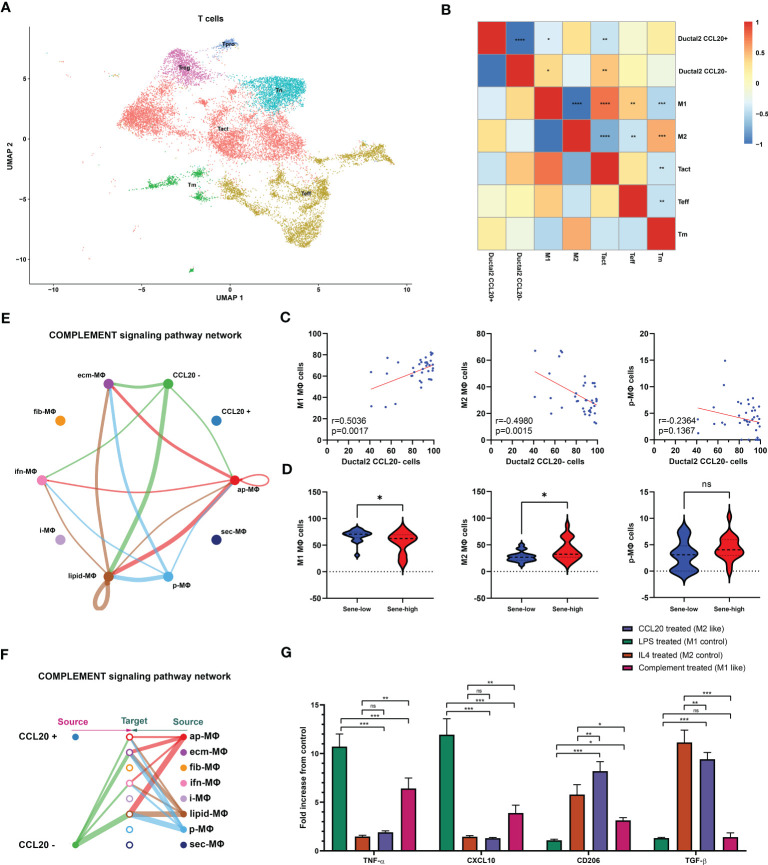
Senescence abrogates complement-mediated M1. **(A)** Further clustering of T cells projected on UMAP into 6 functional states. Correlation between the frequency of specific cell types evaluated by Pearson’s correlation analysis with r value presented as heat map **(B)** and correlation plot **(C)**. **(D)** Comparison of cell type frequencies between sene-low and -high patients. **(E, F)** Number of cellular interactions of the complement signaling pathway between CCL20 + versus - Ductal 2 cells and macrophage subsets inferred by CellChat. **(G)** Characterization of typical M1 products (TNFa and CXCL10) and M2 products (CD206 and TGFb) after stimulation with CCL20, complement protein, compared to control polarization phenotypes of LPS-induced M1 and IL4-induced M2, with expression values normalized to baseline expression in PMA-activated, undifferentiated THP-1 cells. * p<0.05, ** p<0.01, *** p<0.001, ns not significant.

**Figure 7:**
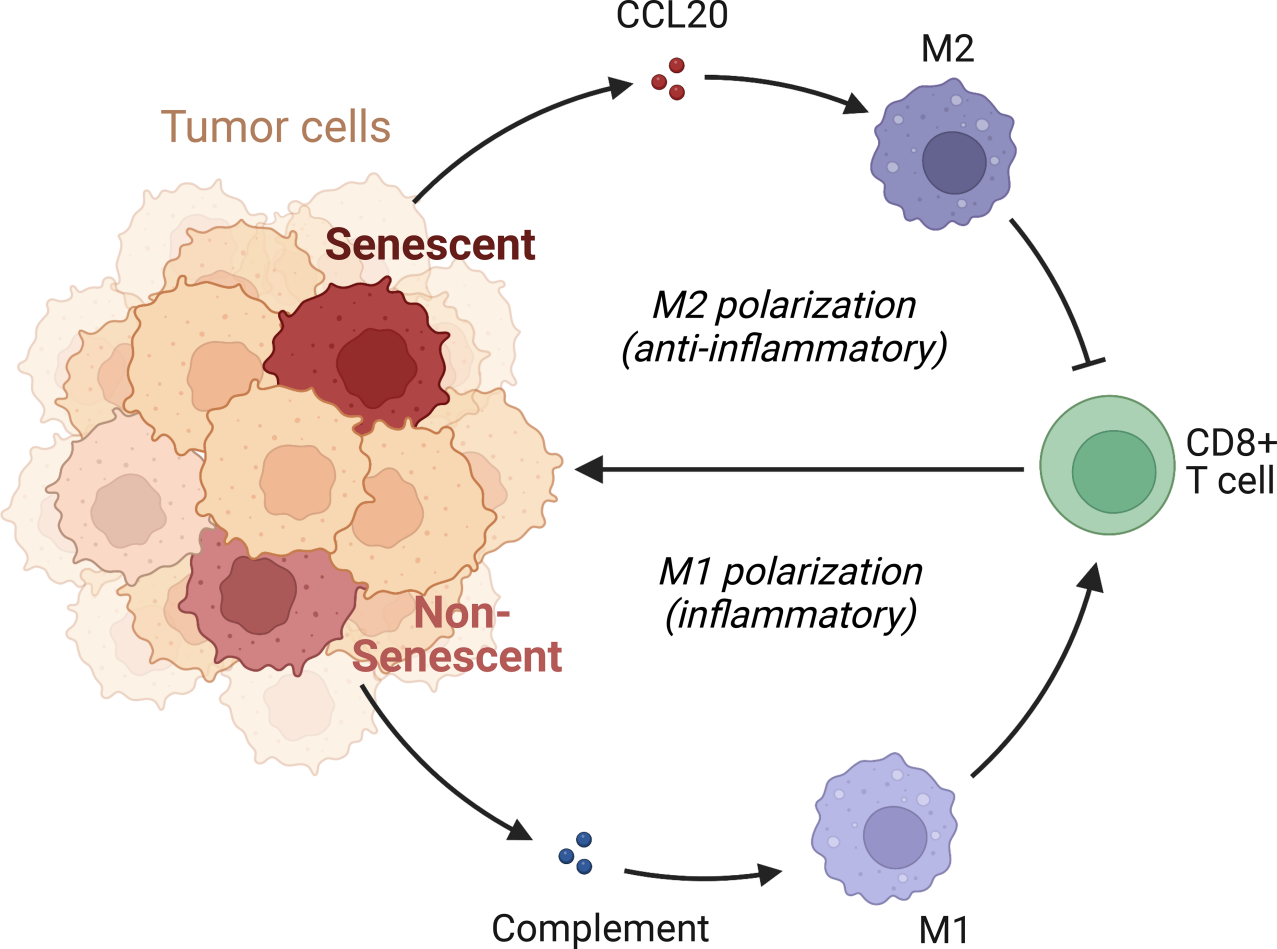
Mechanism of immunosuppression by senescent tumor cells. Senescent tumor cells acquire SASP with enhanced secretion of CCL20, acting on CCR6 receptors of precursor macrophages to favor the immunocompromised M2 phenotype. Non-senescent tumor cells release complement and elicit pro-inflammatory, effective M1 response.

We subsequently investigated the mechanism through which high CCL20 exert immunosuppressive effects. Analyzing cellular interaction between major cell types with CellChat revealed most extensive interaction between ductal 2 and myeloid cells (**Figure 5H**, **Table S4**). Therefore, we further clustered myeloid into eight subsets: fibroblastic (fib), inflammatory (i), secretory (sec), interferon-stimulated (ifn), extracellular matrix producing (eco), lipid metabolizing (lipid), antigen presenting (ap), and precursor (p) macrophages (**Figures 5I, J**). AUCell demonstrated active antigen presentation in lipid-M, ap-M, and ifn-M and ecm-M; while i-M, sec-M, and fib-M had comparatively lower antigen presentation capacity (**Figure 5K**, **Figure S4B**). Based on these, we annotated p-M, inflammatory M1 (lipid-M, ap-M, and ifn-M and ecm-M) and immunosuppressive M2 (i-M, sec-M, and fib-M). To confirm the validity of our annotation in the complex TME with highly dynamic myeloid functional and phenotypical spectrum, we further annotated T cells into proliferating T (Prof), regulatory T (Treg), naive T (Tnaive), activated T (Tact), effector T (Teff), and memory T (Tm) based on conventional markers of T cell functions and highly expressed transcripts specific to each cluster (**Figure 6A**, **Figure S4C**). Notably, M1 macrophages were positively correlated with Tact and Teff, while M2 macrophages are in negative correlation (**Figure 6B**). The cognate receptor of CCL20, CCR6, is ubiquitously expressed in p-M, suggesting the potential effect of CCL20 in macrophage differentiation (**Figure 5J**, **Figure S4D**). Indeed, CCL20+ ductal 2 cells were positively associated with M2 but negatively associated with M1, seeming to favor M2 differentiation over M1 (**Figures 6B–D**). On the contrary, analysis of cellular interactions by CellChat revealed that M1 macrophages were stimulated by complement from CCL20- but not CCL20+ tumor cells (**Figures 6E, F**). *In vitro* experiment in the macrophage cell line THP-1 revealed that CCL20 treatment gave rise to M2-like phenotype with high CD206 and TGFb expression, while complement stimulated M1 polarization and enhanced secretion of TNFa and CXCL10 (**Figure 6G**). In summary, senescent tumor cells secrete CCL20 to induce immunosuppressive M2 polarization, while complement derived from non-senescent tumor cells stimulates M1 antigen presentation and antitumor immune responses (**Figure 7**).

### 3.4 Senescence risk model

**Figure 8:**
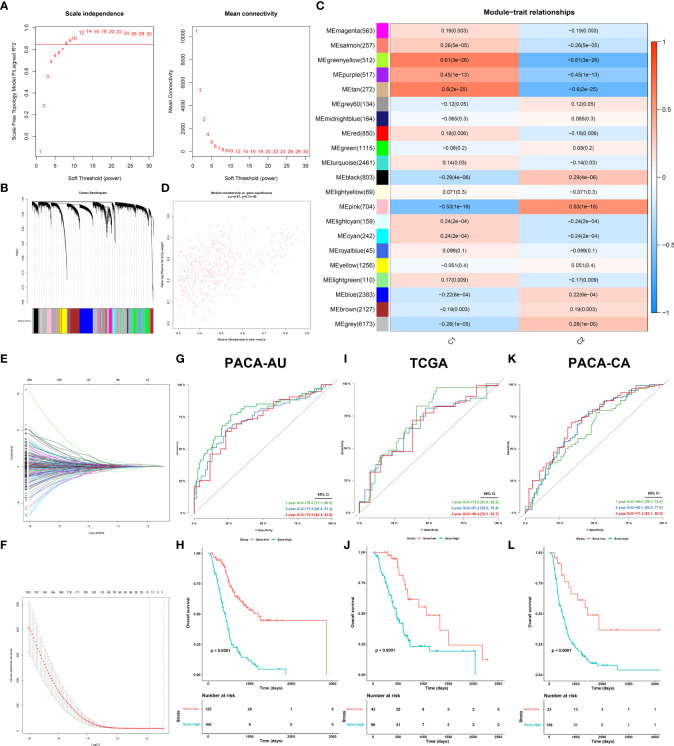
Establishment and validation of senescence-based prognostic signature. **(A, B)** optimization of WGCNA parameters. **(C)** Heat map of correlation between gene modules and C2 characteristic. **(D)** Scatter plot of gene significance of body weight and module membership in the pink module. **(E, F)** LASSO (least absolute shrinkage and selection operator) parameter optimization and selection of the most prognostic genes. Internal validation of the signature in PACA-AU dataset by time-dependent ROC **(G)** for 1-, 2-, and 3-year survival probability and Kaplan-Meier survival analysis **(H)** between sene-low and -high stratified by optimal cut-off value determined from maxstat, External validation of the signature in the TCGA **(I, J)** dataset and PACA-CA **(K, L)** dataset.

We next sought to develop a predictive model with fewer genes for better clinical utility and practicability. Construction of model in PACA-AU. The weighted gene co-expression network analysis (WGCNA) was applied to all genes and parameters adjusted for optimization (**Figures 8A, B**), giving rise to 21 modules by average linkage hierarchical clustering, among which the pink module (704 genes) was the most correlated with C2 (Pearson’s correlation coefficient = 0.53, P < 0.001) (**Figures 8C, D**, **Table 5**). Finally, the LASSO regression algorithm screened out 12 genes to be included in the senescence risk model (**Table S5**, **Figures 8E, F**), with time-dependent ROC curve analysis showing good for OS prediction (1-year AUC = 0.786, 2-year AUC = 0.739, 3-year AUC = 0.725) (**Figure 8G**). Stratification of patients by an optimal cutoff value determined from the maximally selected rank statistics (‘maxstat’ R package) revealed two distinctly separated survival curves on Kaplain-Meier analysis (log-rank test, P < 0.0001; mOS 1142 days versus 413 days, **Figure 8H**). The model is further externally validated in TCGA (1-year AUC = 0.730, 2-year AUC = 0.672, 3-year AUC =0.664, **Figure 8I**) (log-rank test, P < 0.0001; mOS 1037 days versus 466 days, **Figure 8J**) and PACA-CA (1-year AUC = 0.646, 2-year AUC = 0.691, 3-year AUC = 0.715, **Figure 8K**) (log-rank test, P < 0.0001; mOS 1359 days versus 482 days, **Figure 8L**), suggesting validity and robustness of this model.

**Figure 9:**
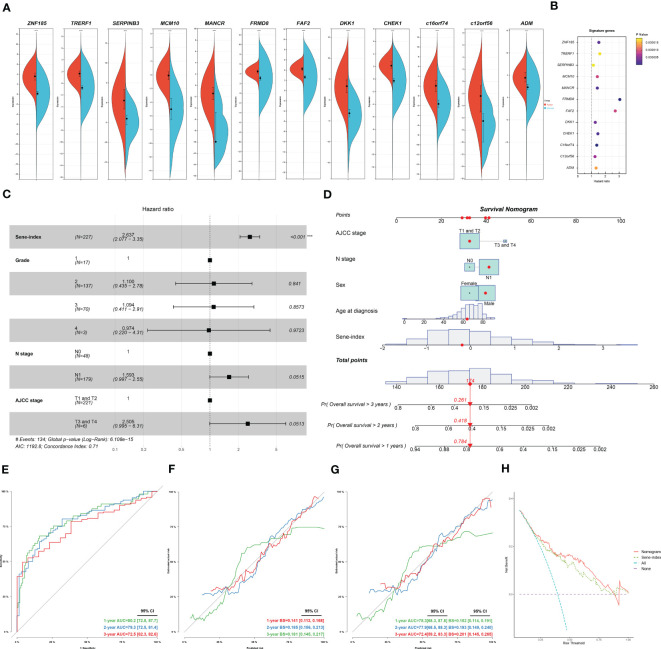
Construction and application of senescence-based nomogram. **(A)** Compare the expression of the 12 genes of the senescence signature between normal and cancerous pancreatic tissue. **(B)** Independent survival analysis of the 12 genes in PDAC. **(C)** Multivariate COX regression analysis of prognostic value of sene-index and clinicopathological parameters, HR and p-values displayed on forest plot. **(D)** Nomogram. **(E, F)** Nomogram calibration for OS-prediction at 1-, 2-, and 3-year. **(G)** Internal validation by bootstrapping (b=100) at 1‐, 2‐ and 3‐year, evaluation by AUC and Brier score. **(H)** DCA comparing the nomogram and the sene-index alone.

We further investigated each of the individual 12 genes included in the model. All genes are unregulated in cancerous versus normal pancreas tissue (**Figure 9A**). Individual survival analysis revealed hazard ratios all bigger than 1 (**Figure 9B**). The potential biological significance of each gene is further explored in the discussion section. Furthermore, we used univariant Cox regression to identify all OS-related characteristics with significant regression coefficients and p-values (**Table S5**), and then used multivariate Cox regression to determine that Sene-index is an independent and robust prognostic factor (HR, 2.637; 95% CI, 2.077–3.350; P < 0.001; **Figure 9C**). We then constructed a nomogram containing all prognostic characteristics, specifically the Sene-index, grade, N stage, and AJCC stage (**Figure 9D**). The nomogram had accurate predictive power for the survival for pancreatic cancer patients at 1 year (AUC=0.802, Brier Score=0.141), 2 year (AUC=0.793, Brier Score=0.185), and 3 year (AUC=0.725, Brier Score=0.181) (**Figures 9E, F**). Further validated by internal validation with bootstrapping (b=100), at 1‐, 2‐ and 3‐year (**Figure 9G**). 1 year (AUC=0.783, Brier Score=0.152), 2 year (AUC=0.779, Brier Score=0.193), and 3 year (AUC=0.724, Brier Score=0.201). The DCA dataset demonstrated that this nomogram performed better than sene-index alone in predicting the 1‐, 2‐ and 3‐year survival of patients with pancreatic cancer (**Figure 9H**).

### 3.5 Senescence and drug resistance

**Figure 10:**
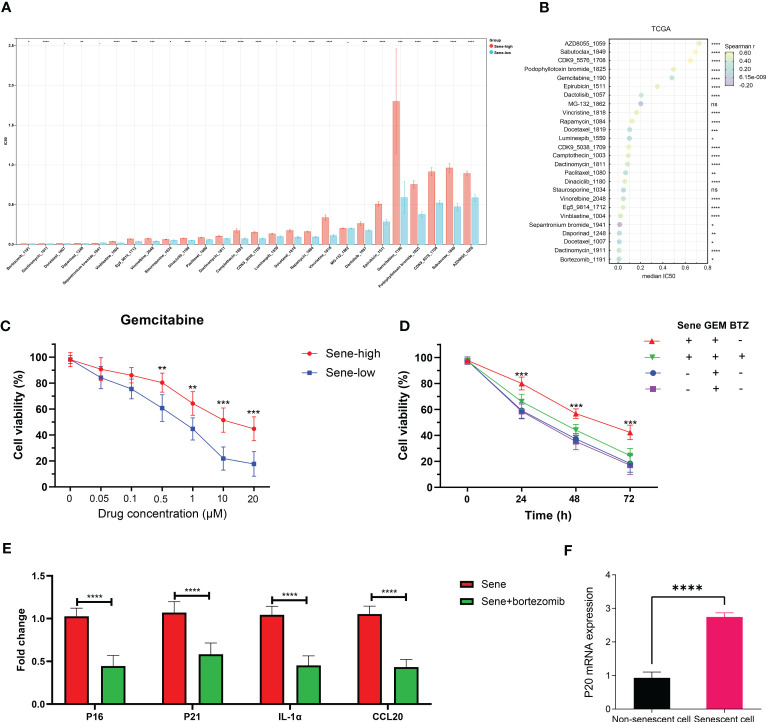
Proteasome inhibitors reverse chemoresistance conferred by senescence. IC50 of chemotherapeutic drugs in TCGA PDAC patients was predicted by OncoPredict and compared between sene-high and -low **(A)** and correlation analysis with sene-score **(B)**. **(C)** Control and doxorubicin-induced senescent PANC-1 cells were treated with gemcitabine of various concentration (0, 0.05, 0.1, 0.5, 1, 10, and 20 uM) for 24 hours and cell viability evaluated with MTT assay. **(D)** PANC-1 cells are with or without doxorubicin-pretreatment to induce senescence are challenged with gemcitabine alone or gemcitabine + bortezomib for 24 hours before evaluation of cell viability by MTT assay. **(E)** RT-qPCR quantification of mRNA expression of senescent genes (p16, p21,IL1a, and CCL20) in doxorubicin-pretreated senescent PANC-1 cells with or without bortezomib treatment for 24 hours, using non-senescent PANC-1 as control. **(F)** RT-qPCR quantification of mRNA expression of proteasome subunit p20 in control versus senescent PANC-1 cells. * p<0.05, ** p<0.01, *** p<0.001, **** p<0.0001, ns not significant.

The Oncopredict algorithm of drug response prediction revealed resistance to most drugs in the Sene-high subtype (**Figure 10A**) and significant correlation between senescence status and IC50 (**Figure 10B**). Senescence seems to confer resistance to the two most commonly used chemotherapeutic drugs in pancreatic cancer patients, gemcitabine more than paclitaxel, as validated in pancreatic cell line with radiation-induced senescence (**Figure 10C**). In light of the seemingly low IC50 of bortezomib and the lack of resistance to MG132 when comparing high and low senescence patients, proteasome inhibitors are considered to have potential therapeutic value for the treatment of senescent pancreatic cancers, as validated that bortezomib inhibit the long-term persistence senescent cells in conjugation with gemcitabine significantly in senescent cells but not normal (**Figure 10D**). We sought to further elucidate the role of senescence by defining the status of cellular senescence with biomarkers, identifying up-regulated mRNA expression of p16, p21, SASP IL1a and CCL20 in senescent cells by bortezomib treatment (**Figure 10E**). As proteasome inhibition disrupted senescence, we confirmed the importance of proteasome function in allowing senescence, as p20 is highly expressed in senescent cells rather than normal cells (**Figure 10F**).

## 4 Discussion

PDAC has long been a challenging disease, with high death rate ranking the fourth in place despite a relatively low frequency of occurrence (34). Patients with PDAC only saw marginal benefits from the advancements in chemotherapeutic agents and immunotherapy that drastically improved the survival of many other cancer types over the last decades. Attempting experimental therapies is especially important for patients with PDAC. However, it is important to keep in mind that each patient’s tumor may have different biological characteristics, rendering them to respond differently to the same therapy. Therefore, going beyond the traditional classifications based on clinicopathological characteristics and even molecular or microenvironment traits, to develop new models of patient stratification based on activities of biologically meaningful pathways, would help identify the key problem of each tumor and allow specific therapeutic assignment accordingly, achieve better response and bring precision medicine into reality.

Cellular senescence is conventionally considered the permanent arrest of cell cycle and inhibition of cell division, seemingly a tumor suppressive mechanism as the solution to stop the rapid growth of tumor cells (35). The senescence-related effects of conventional chemotherapies and radiotherapies, especially DNA-damaging mechanisms, have been well reported, suggesting senescence at low dose but apoptosis at higher dose (36, 37). However, newer opinions agree that cellular senescence is a potentially reversible process of dynamic epigenetic and transcriptional remodeling, often exploited by tumor cells for drug resistance, resistance to stress-induced cell death, and immune-evasion. In clinical settings, senescence has multimodal functions in different cancers: our previous study identified hazardous role of senescence in breast cancer (38), Zhou et al. reported that senescence is associated with good survival in gastric cancer (39), but Dai et al. reported opposite findings (40). This study supports the role of tumor senescence as a hazardous factor, associated with significantly shorter survival outcome, immunosuppression and therapeutic resistance in patients with PDAC.

Mechanistically, our study suggested that in PDAC, senescence is: (1) paradoxically associated with more cell division; (2) extensive suppression of immune activity leading to a high-purity TME. Going over the key components of an immune response, we discovered that despite comparable antigenicity (if not more antigenic due to a higher TMB) of senescent tumors, APCs activation, function and antigen presentation are impaired. SASP refers to the specific secretory phenotypes of senescent cells. Given the dynamic, context-dependent expression patterns of SASP factors with broad functional ranges, we next identified CCL20 as the key SASP factor mediating immunosuppression in senescent pancreatic tumor, with its cognate receptor CCR6 highly expressed on naive, precursor macrophages, to mediate M2 polarization and immunosuppression. Also, senescent status seems to decrease the release of immunogenic, pro-M1 complement proteins from tumor cells, further enhancing immunosuppression. The dual potential of in macrophages cancer were recently well reviewed, reflecting their plasticity in response to environmental cues (41). In breast cancer, tumor-derived CCL20 has been recently reported to signal on CCR6 in precursor macrophages, promote PMN-MDSC expansion and exert pro-tumor effects (42). In osteosarcoma, complement-associated macrophages have been reported to mediate M1 polarization and antitumor immunity (43). However, the role of CCL20-stimulated M2 and complement-stimulated M1 remain unreported in pancreatic cancer, a comprehensive consideration in the context of senescence is also missing. These studies in other cancer types provide further support to our conclusions, as the underlying biological mechanisms and subtype-specific cellular phenotypes are shared despite differences between cancer types.

We next attempted to translate these senescence-related discoveries to clinical practice. A significant limitation of the TNM staging system is that it does not account for biologic factors with predictive and prognostic value (44). To address it, a risk model was constructed to stratify patients with pancreatic cancer into high- and low-risk subgroups with different senescent statuses. Survival analysis, ROC curves and calibration covers all confirmed the predictive capacity of this model at different timepoints for the outcome of patients with pancreatic cancer. The model is also validated in multiple individual datasets. For high-risk patients identified through our model, we attempted to identify potentially beneficial therapeutic improvements with the Oncopredict algorithm. In line with other studies reporting that cellular senescence is key to chemoresistance (45), we also found out that most drugs have a higher IC50 value in the high-risk group, especially the most used chemotherapeutic agent in pancreatic cancer, gemcitabine, with the mechanism of inhibiting DNA synthesis through dFdCTP incorporation in S phase. Senescence-mediated resistance to DNA-damaging chemotherapy is further confirmed *in vitro* cellular experiment. On the other hand, the microtubule-targeting agent paclitaexl, also commonly used for patients with PDAC, is considerably less affected by senescence, suggesting potential benefit in choosing paclitaxel-based therapy over gemcitabine-based regime for these high-risk patients. In addition, we discovered that metabolic drugs such as proteasome inhibitors seem to have comparable IC50s between the two groups unaffected by senescence. The therapeutic value of bortezomib was further confirmed in senescent tumor cells but not regular, suggesting the dependency of senescent cells on proteasome function.

As the data based on which we built our model was obtained from public databases and all patients retrospectively enrolled, we acknowledge the insufficiency in our study in the absence of prospective validation and further support by prospectively generated transcriptomic data. Our model, as a risk score calculation based on 12 genes, have the advantage of cost-effectiveness, practicability, and ease of clinical implementation. Further validation on the protein level with preserved pathological samples or prospective, observatory clinical trial in patients with PDAC would provide better power to the value of this model. Furthermore, despite seemingly clear mechanistic studies, the therapeutic effects and actual gains from the addition of proteasome inhibitor, namely bortezomib, onto conventional chemotherapies in the high-risk patients based on the result of the stratification awaits further confirmation. Preliminary *in vitro* results in senescent cell lines demonstrated promising results in the ability of bortezomib to reverse senescence and gemcitabine-resistance, and supports from *in vivo* experiment in xenograft or PDX mice models would provide more confidence along the line to clinical translation.

## 5 Conclusions

In this study, we identified senescence in PDAC as tumor-specific, hazardous factor associated with immunosuppression. A senescence-based model stratified patients with PDAC into two populations with distinct clinical outcomes and therapeutic opportunities. Mechanistic studies revealed that non-senescent tumor cells release complement proteins to differentiate macrophages toward M1 phenotype with T cell stimulatory capacities. This process is impaired in senescent tumor cells, which secrete CCL20 to favor M2 polarization and to abrogate antigen presentation, leading to an immune desert microenvironment. Interestingly, the senescent phenotype is highly dependent on proteasome functions, suggesting potential value proteasome inhibitors in high-risk patients with senescent pancreatic tumors to overcome resistance to conventional chemotherapy and immunotherapy, ultimately improving outcome through precision medicine.

## Data availability statement

The original contributions presented in the study are included in the article/**Supplementary Materials**, further inquiries can be directed to the corresponding author/s.

## Author contributions

Conception and design: ZL, MW. Development of methodology: MW, JH. Analysis and interpretation of data: MW, HW. Writing of the manuscript: JH. Review of the manuscript: ZL. All authors contributed to the article and approved the submitted version.
