# Supplementary figures and images for: Proteasome-dependent senescent tumor cells mediate immunosuppression through CCL20 secretion and M2 polarization in pancreatic ductal adenocarcinoma
Source: Front Immunol. 2023 Jun 15;14:1216376. doi: 10.3389/fimmu.2023.1216376 (PMC10310997; doi:10.3389/fimmu.2023.1216376)

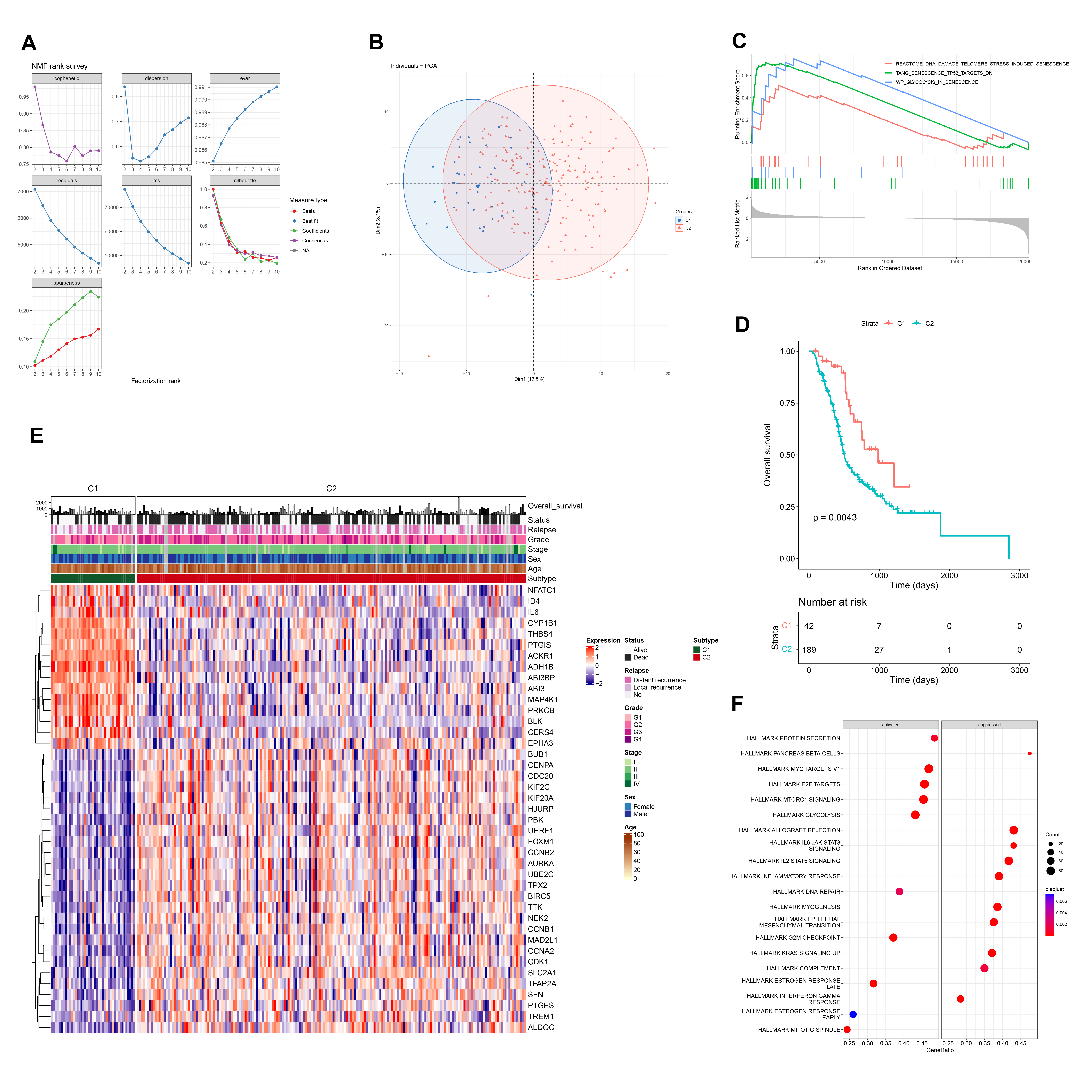

Supplement: Supplementary file 1 [file DataSheet_1.zip › Supplementary Materials/Figure S1.tif]

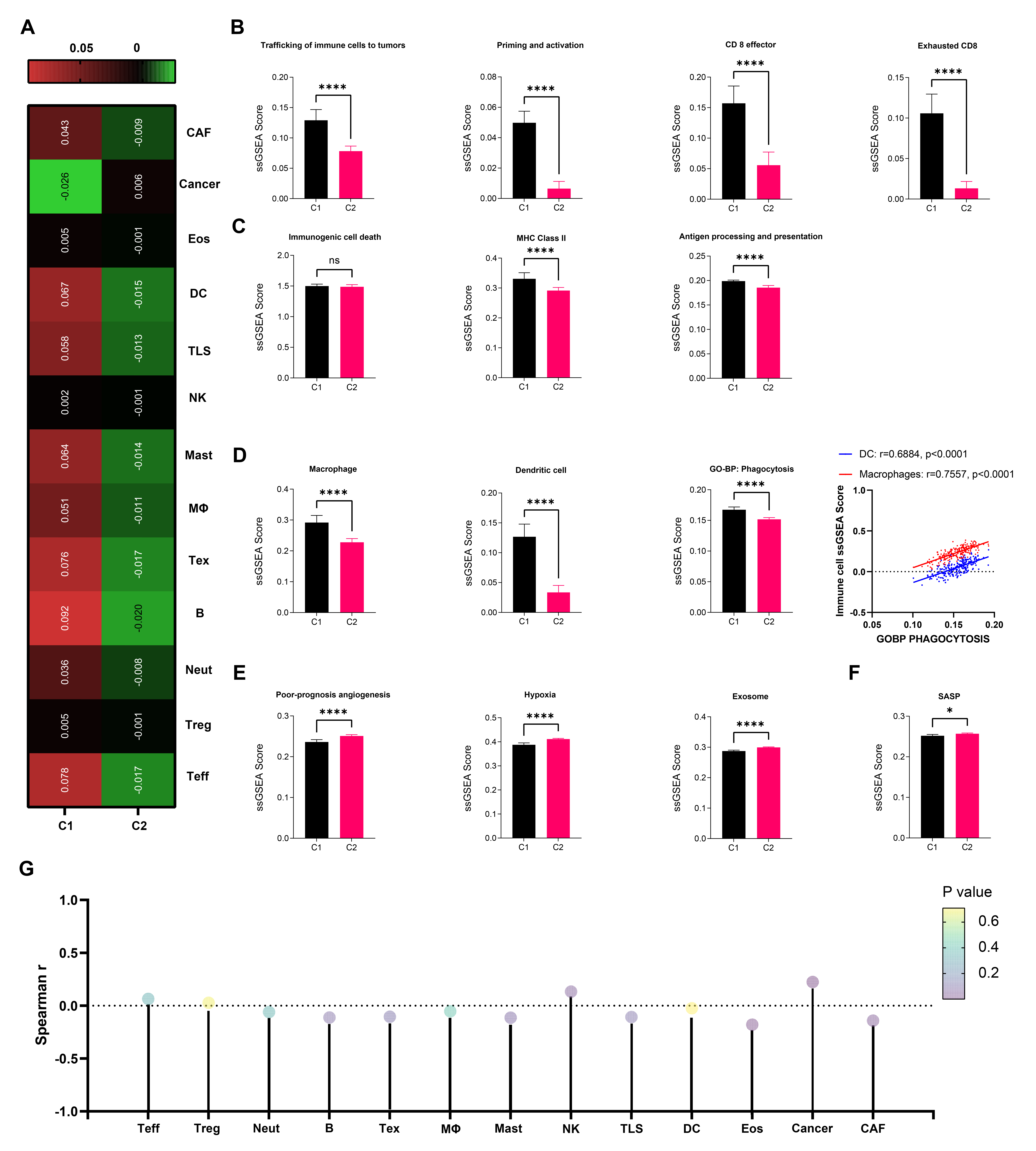

Supplement: Supplementary file 1 [file DataSheet_1.zip › Supplementary Materials/Figure S2.tif]

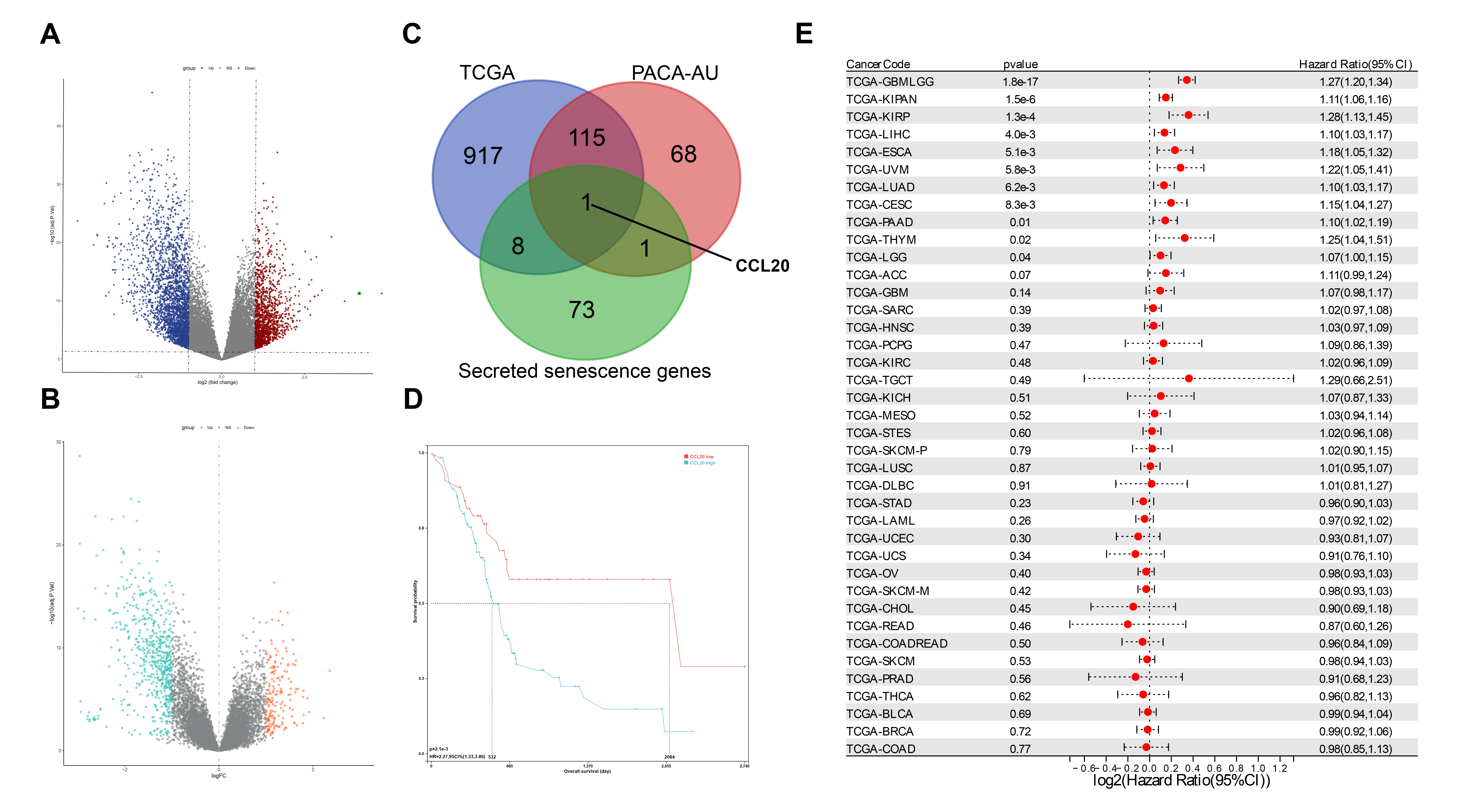

Supplement: Supplementary file 1 [file DataSheet_1.zip › Supplementary Materials/Figure S3.tif]

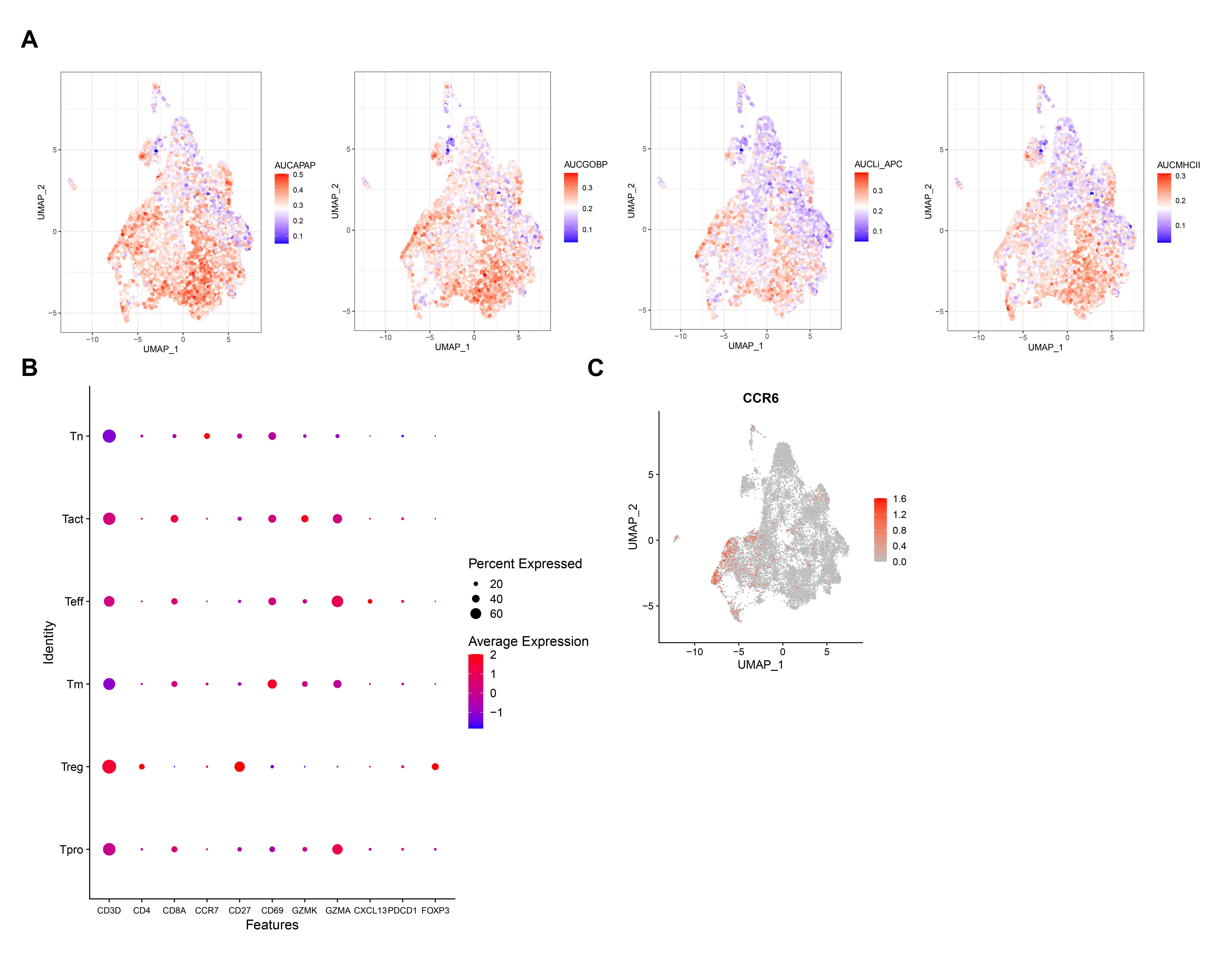

Supplement: Supplementary file 1 [file DataSheet_1.zip › Supplementary Materials/Figure S4.tif]
